# Primary Anaplastic Ganglioglioma of the Temporal Lobe With Brainstem Involvement: A Case Report and Literature Review

**DOI:** 10.7759/cureus.12060

**Published:** 2020-12-13

**Authors:** Artsiom Klimko, Mariana Dandes, Francesca Paslaru, Andrei Giovani

**Affiliations:** 1 Division of Physiology and Neuroscience, University of Medicine and Pharmacy "Carol Davila", Bucharest, ROU; 2 Department of Neurological Surgery, Emergency Clinical Hospital "Arseni-Bagdasar", Bucharest, ROU

**Keywords:** anaplastic ganglioglioma, recurrence, pontine invasion, malignant glioma, brainstem

## Abstract

Anaplastic ganglioglioma (AGG) is a rare and aggressive counterpart of the more benign and frequently encountered glioma. Herein, we present a 21-year-old female who presented with episodes of total amnesia and complex partial seizures, which led to the diagnosis of AGG localized to the medial temporal lobe. She subsequently underwent surgical cytoreduction of the tumor three times with adjuvant chemoradiotherapy. The extent of resection throughout the surgeries was hindered by the extension of the tumor to critical neurovascular structures; during the last surgery, invasion into the pons was noted, which posed a significant clinical challenge.

## Introduction

Gangliogliomas are uncommon mixed neuronal-glial tumors, representing 1.3% of brain tumors and 0.4% of central nervous system neoplasms [1]. The majority of these tumors are encountered as low-grade gangliogliomas, although malignant transformation, especially in patients who underwent subtotal resection and underwent radiotherapy, has been reported [2]. In rare circumstances, as in this case report, primary high-grade anaplastic gangliogliomas (AGG) can arise spontaneously and cause significant patient morbidity. The median overall survival (OS) can be as low as 24.7 months, making AGG one of the most aggressive entities in neurological oncology [3-4]. Gangliogliomas have a predilection for supratentorial locations, especially the temporal lobe, where they are highly epileptogenic, being a common cause of medically refractory lesional epilepsy [5].

We present a case report of a young female with a recurrent primary anaplastic intracranial ganglioglioma, which was repeatedly treated with surgical cytoreduction and adjuvant therapy. As neurosurgeons are unlikely to see more than just a few cases during their practice, we hope to detail what type of outcome trajectory can be expected in the circumstances when gross-total resection (GTR) of an AGG is not feasible. We further conducted a literature review to describe patient characteristics and survival outcomes in patients with AGG and brainstem involvement.

## Case presentation

**Figure 1:**
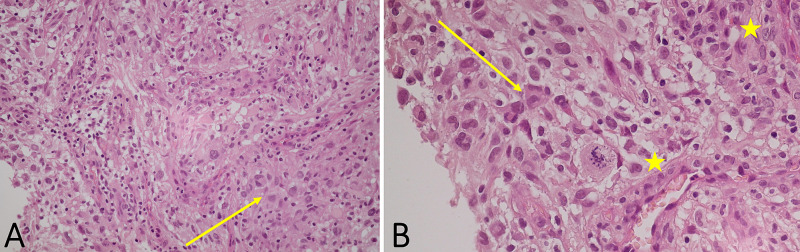
Histopathologic analysis Image A (H&E, x200) showing a haphazard arrangement of abnormal glial cells (yellow arrow); image B (H&E, x400) showing glial cells with abnormal, enlargement nuclei (yellow arrow), microcysts, and perivascular lymphocytic cuffing (yellow stars) suggestive of anaplastic ganglioglioma H&E: hematoxylin and eosin

A 21-year-old female patient was hospitalized in our neurosurgical department with episodes of total amnesia and complex partial seizures, lasting two to three minutes and occurring as often as seven times per day. Throughout the seizures, the patient didn’t lose consciousness and the onset of symptoms occurred gradually over two months. Further investigations revealed an expansive mass in the right medial temporal lobe - resection was attempted, but somewhat hindered due to adhesion of the superior pole of the tumor to the branches of the posterior cerebral artery (PCA). Histopathologic analysis of the resected specimen demonstrated dysplastic glial proliferation, with abnormal appearing ganglion cells (Figure 1). The preliminary pathology report suggested a diagnosis of anaplastic xanthoastrocytoma, which was later revised to AGG. The patient had an uneventful postoperative course, marked by resolution of the inceptive seizures and normal postoperative imaging. The patient was subsequently discharged and referred to oncology where her treatment was supplemented by an adjuvant six-month chemotherapy regimen, consisting of temozolomide and carboplatin, and 3D conformal radiotherapy administered over 30 daily fractions for a total dose of 60 Gy in four weeks.

**Figure 2:**
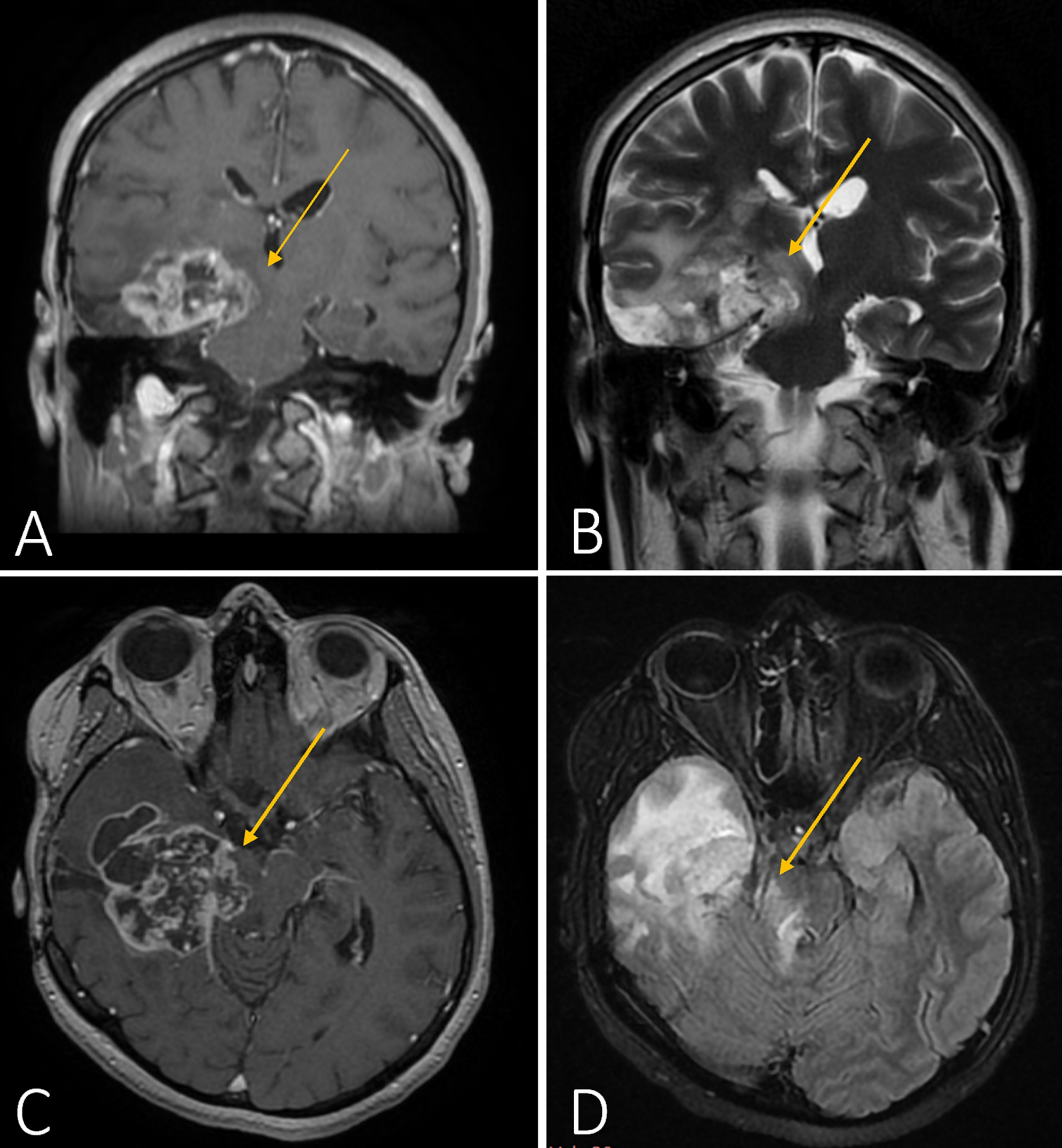
Cranial MRI prior to the third surgery Coronal (images A and B) and axial (images C and D) T1- and T2-weighted images, respectively, showcasing right-sided pontine extension of the tumor (yellow arrows) MRI: magnetic resonance imaging

The patient was readmitted five months later with left-sided hemiparesis caused by tumor recurrence. Surgical cytoreduction was conducted by extending the resection beyond the medial ambient cistern, however, a small fraction of the tumor remained due to its extension around the PCA. Postoperative recovery was uneventful and after discharge, a six-month salvage chemotherapy protocol of temozolomide and bevacizumab was started. An intractable headache, left hemiparesis, and visual disturbances lead to the third re-admission of the patient to our neurosurgical department 12 months later. Imaging revealed a tumor recurrence localized to the right temporal lobe; however, at this time, extension into the right margin of the midbrain and pons was noted (Figure 2).

**Figure 3:**
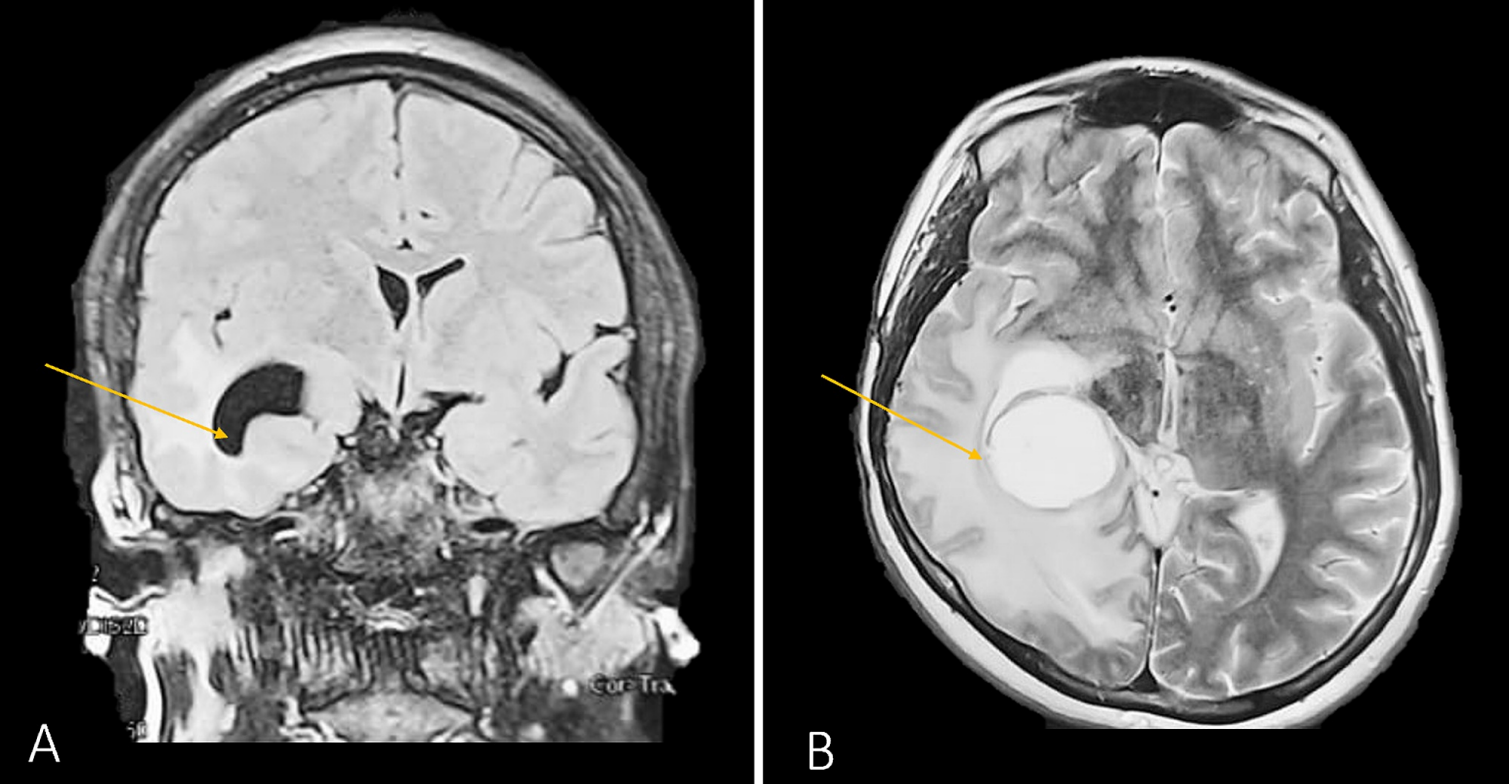
Cranial MRI after the third surgery Coronal (image A) and axial (image B) T2-weighted images, showcasing a right-sided partial lobectomy with sparing of the superior temporal gyrus (yellow arrows) MRI: magnetic resonance imaging

A subtemporal craniotomy with extended margins was done to conduct the third resection. Intraoperatively, the tumoral bulk appeared friable and well-vascularized. Massive superior and inferior extensions of the tumor necessitated a partial temporal lobectomy, which excluded the superior temporal gyrus. GTR was again hindered by extensions that were in close proximity to the PCA, the basal vein of Rosenthal, and the pons. Due to the concern of precipitating devasting neurologic sequelae, resection of the tumor from these structures was not attempted; the extent of resection is shown in Figure 3. Postoperatively, the overall status of the patient improved and the motor deficit and seizure control improved. Unfortunately, visual disturbances (in the form of left-sided quadrantanopia) persisted, presumably due to the invasion of the tumor into Meyer’s loop. Chemotherapy with temozolomide and bevacizumab was resumed. Upon follow-up six months later, the patient’s status is favorable - seizures have not recurred, she is able to ambulate without assistance, and the disease appears stable.

## Discussion

In this case report, we presented a patient who underwent surgical cytoreduction three times for a primary anaplastic intracranial ganglioglioma. During the last surgery, brainstem involvement was noted. In a retrospective analysis of 326 primary intracranial gangliogliomas, Blumcke et al. found only 17 cases that were classified as anaplastic (grade III) gangliogliomas [6]. By cell type, this tumor consists of dysplastic neuronal and neoplastic gliomatous components; the degree to which these components are expressed determines the diagnosis. Histopathologic analysis can be challenging, as we encountered in our case because the biphasic morphology can be differentially expressed with other pleomorphic components like desmoplastic gangliocytoma or xanthoastrocytoma [7-8].

A central theme of high-grade gangliogliomas is their tendency to recur. In a large retrospective series of 184 supratentorial gangliogliomas, the recurrence rate of first, second, and third-grade gangliogliomas was 1%, 18%, and 50%, respectively [2]. Treatment consisted of surgical resection with chemoradiotherapy for patients diagnosed with AGG and although no information was provided regarding the median extent of resection (EOR) values, most patients (79%) did not have a residual tumor on postoperative evaluation. Another study examining 58 gangliogliomas reported higher recurrence rates (33%), even after radical resection [9]. The relative risk for recurrence was five-fold higher for tumors affecting the brainstem, compared to a more typical hemispheric location. Another series of 53 gangliogliomas quantified the benefit that surgical cytoreduction on recurrence-free survival and observed a significantly improved recurrence-free survival rate if EOR exceeds 94% [10]. However, in all of these studies, only a minority of examined tumors were AGG, and thus, small sample sizes precluded the authors from conducting a multifactorial analysis specifically for high-grade gangliogliomas.

Due to the rarity of AGG, evaluation of the Surveillance, Epidemiology, and End Results (SEER) cancer registry has been invaluable in detailing epidemiology and prognostic factors. In a large series of 58 tumors and a median follow-up period of 52 months, Selvanathan et al. found the median OS and five-year survival rates for AGG to be 28.5 months and 63%, respectively [3]. AGG was most commonly diagnosed in males under 40 years of age, arising in the temporal and frontal lobes; other findings are summarized in Table 1. The study concluded that multifocal tumors and the inability to achieve GTR to be predictors of survival. Although radiotherapy is widely used as adjuvant treatment for grade I gangliogliomas and there was a trend for prolonged survival in patients with AGG who received adjuvant radiotherapy, the difference was not statistically significant. The crucial limitations of this study included a lack of information regarding EOR, tumor size, and type/dosages for adjuvant therapy that was administered.

**Table 1:** Patient and tumor characteristics of anaplastic ganglioglioma as compared to our patient FLAIR: fluid-attenuated inversion recovery; NA: not applicable

Patient and tumor characteristics	Most common findings in the study by Selvanathan et al., 2011 [3]	Most common findings in the study by Terrier et al., 2017 [4]	Our case
Age	16-39 (47.2%)	30-39 (27.9%)	21
Sex	Male (70.0%)	Male (60.5%)	Female
First symptom	NA	Seizures (37.2%)/focal deficit (37.2%)	Seizures and amnesia
Tumor location	Temporal lobe (26.6%)	Frontal lobe (37.2%)	Temporal with brainstem involvement
Number of primaries	1 (92.9%)	1 (94.7%)	1
Surgery	Yes (92.9%)	Yes (95.3%)	Yes
Average diameter	NA	46.2 mm (contrast) and 70.3 mm (FLAIR)	52.7 mm (contrast) and 65.4 mm (FLAIR)
Radiotherapy	No (64.2%)	Yes (27.9%)	Yes

The French Brain Tumor Database study by Terrier et al. attempted to further understand the biological behavior of AGG by providing more information regarding imaging characteristics, treatment strategy, and recurrence statistics [4]. A total of 43 patients were reviewed retrospectively. The median follow-up period was 42.5 months while the median overall survival (OS) and five-year survival rates were 24.7 months and 24.9%, respectively. The significant discrepancy in five-year survival rates between this study and the SEER study by Selvanathan et al. is likely due to the exclusion of pediatric cases from the latter investigation, as the median OS for pediatric patients who underwent GTR can exceed 36.5 months [3,11]. GTR was achieved in 20 of 34 patients who opted for surgical treatment, and tumor location in the frontal lobe or midline crossing was associated with significantly shorter OS. The therapeutic protocol with the highest median OS (37.03 months) consisted of GTR with adjuvant chemoradiotherapy per the protocol by Stupp et al. while patients who underwent subtotal resection did not benefit from combined chemoradiotherapy [12].

To further explore AGG brainstem involvement, a search of the PubMed and Scopus databases was done using the keywords “anaplastic ganglioglioma,” “brainstem,” “mesencephalon,” “pons,” “medulla,” and “midbrain” to identify relevant case reports. The keywords were identified as either Medical Subject Heading (MeSH) terms or within the title/abstract. A total of 29 studies were found, 22 studies were excluded due to being unrelated, resulting in six case reports and one study being included in this review. The results are presented in Table 2.

**Table 2:** Cases in the literature describing anaplastic ganglioglioma with brainstem involvement, including our patient CPA: cerebellopontine angle; GTR: gross total resection; NA: not applicable

Author and year	Age and sex	Duration of symptoms	Symptoms	Tumor location	Treatment modality	Clinical outcome
Our patient	Female, 23-year-old	Two months	Intractable headache, left hemiparesis, and visual disturbances	Right temporal lobe, midbrain, and pons	Subtotal surgical resection with chemoradiotherapy	Patient doing well during five-month follow up visit
Dutta et al., 2018 [13]	Female, 32-year-old	Three months	Decrease in visual acuity, vomiting, and right-sided hearing loss with tinnitus	Right CPA	GTR with postoperative radiotherapy	Discharge with improved status, no follow up
Boissonneau et al., 2016 [14]	Male, 35-year-old	Five days	Vertigo, headache, and hearing loss	Right CPA	Subtotal resection with adjuvant chemoradiotherapy	Recurrence 18 months later with fatal carcinomatous meningitis
Rusiecki et al., 2017 [15]	Female, 9-year-old	Several weeks	Headache and right-sided hand weakness	Left-sided basal ganglia, midbrain, and pons	Subtotal resection with adjuvant chemoradiotherapy	Deceased eight months after surgical resection
Toledo et al., 2012 [16]	Male, 33-year-old	Two years	Headache and right-sided weakness and contractures	Left-sided tegmental pons, midbrain, and subthalamic region	NA	NA
Karremann et al., 2008 [11]	Male, 14-year-old	Two months	Vomiting and ataxia	Bilateral pons and 4^th^ ventricle extension	Subtotal resection with adjuvant radiotherapy	Upon follow up eight months after surgical resection patient had stable disease
Mutsuzaki et al, 2005 [17]	Female, 64-year-old	Two weeks	Right-sided hearing loss and truncal ataxia	Right-sided CPA	Subtotal resection and 8 months after, palliative radiotherapy	Tumor recurrence was noted eight months after surgical resection; four months later, the patient gradually fell and passed away from respiratory failure
Hirose et al., 1992 [8]	Female, 12-year-old	Two years	Headache, gait disturbance, and right hemiplegia	Right medulla, cerebral peduncle, and upper cervical spinal cord	Subtotal resection, followed by chemoradiotherapy	The patient responded poorly to chemoradiotherapy and passed away eight months after surgical resection

Of the eight patients reviewed, the average age was 27.8 years (range 9-64), with five patients being female and three male. The clinical presentation included classic symptoms of intracranial neoplasms; however, symptom duration was strikingly variable, ranging from two years to several days. In five cases, brainstem involvement was noted on the right side, two left-sided cases, and one case with bilateral brainstem invasion. The average dimensions of the tumor were 46.0x41.6x50.7 mm, although it must be noted that only three cases reported tumor size and from this small sample size, it is difficult to identify if AGG has a predilection for a certain region of the brainstem. In one case, the authors noted atypical neurocytoma to act as a tumor precursor to the AGG [15]. The standard-of-care treatment was based on subtotal surgical resection followed by chemotherapy with temozolomide (90 mg/m^2^) and radiotherapy, ranging from 30-60 Gy. In our patient, bevacizumab was added, as it was shown to have some activity in patients with recurrent high-grade gliomas. Of eight patients reviewed, only one case was amenable to GTR. The risk of tumor recurrence or poor response to adjuvant therapy, expectedly, remains a crucial contributing factor that makes survival past eight months recherché.

There are currently no studies in the literature that examine the efficacy of mitogen-activating protein kinase pathway (MAPK) inhibitors (or their downstream counterparts: B-Raf and mitogen-activated protein kinase). MAPK inhibitors represent a promising systemic treatment modality for AGG and, more recently, case reports began to emerge detailing their potential utility as an adjuvant [18-19]. The factor that influenced survival was small tumor size or disease amenable to GTR. In our literature review, only one case reported the B-Raf V600E mutation status, which was negative.

## Conclusions

Subtotal resection of AGG portends a poor relapse-free survival. The propensity for the recurrence of AGG must be carefully taken into consideration if surgical cytoreduction of the tumor will be undertaken. In rare cases, AGG may extend into the brainstem or other critical neurovascular structures, thus precluding GTR. In spite of limited data from the literature, attempting maximal safe resection followed by vigilant surveillance from disease progression is an accetable therapeutic strategy. The survival benefit posed by adjuvant chemoradiotherapy for patients who underwent subtotal resection is unclear, despite being widely used.
